# Long-Term Outcomes of Articular Surface Replacement (ASR) Implant in Hip Arthroplasty: A Single Institution Review

**DOI:** 10.7759/cureus.36029

**Published:** 2023-03-11

**Authors:** Jin Chuan Yuen, Hee Nee Pang, Yew Lok Woo, Ngai Nung Lo, Darren Tay Keng Jin, Shi Lu Chia, Seng Lin Yeo

**Affiliations:** 1 Department of Orthopaedics, Hospital Tengku Ampuan Afzan, Kuantan, MYS; 2 Orthopedics, Singapore General Hospital, Singapore, SGP; 3 Orthopedics and Traumatology, Singapore General Hospital, Singapore, SGP; 4 Orthopedic Surgery, Singapore General Hospital, Singapore, SGP

**Keywords:** asian variant, long term follow up after orthopedic surgery, total hip replacement (thr), metal-on-metal total hips, depuy asr hip

## Abstract

Various metal-on-metal (MoM) total hip replacements (THRs) have been found to have high short-term failure rates due to adverse responses to metal debris (ARMD). As a consequence, several low-performing THRs have been removed off the market. The purpose of this research was to look at the at least five-year outcomes of patients who had MoM hip arthroplasty at our institution.

In one specialised centre between 2007 and 2008, 24 Articular Surface Replacement (ASR^TM^, DePuy, Warsaw, IN, USA) MoM THRs (in 24 patients, mean age: 56.4 years) were implanted. DePuy ASR hip prosthesis for osteoarthritis or hip fractures were employed in the THR system. All patients were summoned back for a clinical assessment, and imaging was done as needed. The average period of follow-up was 8.0 years (6.0-10 years). In all, eight instances (33.3%) were discovered to have pseudotumors, four hips (16.7%) were revised, and one (4.1%) was operated for ARMD. The Western Ontario and McMaster Universities Arthritis Index (WOMAC) and Oxford ratings improved statistically significantly five years after surgery in all three areas of pain, disability, and stiffness; however, there was no statistically significant change in the 36-Item Short Form Survey (SF-36) (mental) score. MoM hip arthroplasty had a greater revision incidence at five years in our group, presumably owing to the adoption of a smaller femoral head size.

## Introduction

Although total hip arthroplasty (THA) has been shown to be a predictable therapy with excellent long-term outcomes, changing patient activity levels, demands, and expectations are pushing the need for implants that are more durable and last longer. The subject of which childbearing pair is best is still up for debate. Despite the availability of long-term data demonstrating low rates of revision and osteolysis for metal-on-conventional uncrosslinked polyethene bearing surfaces [1-3], the changing demographic of hip arthroplasty patients drives the pursuit of improvements in bearing surfaces in order to extend implant longevity. Although long-term data are not yet available, innovative bearing combinations such as metal or ceramic-on-highly-cross-linked-polyethene, ceramic-on-ceramic, and metal-on-metal (MoM) implants are expected to outperform traditional metal-on-conventional uncross-linked polyethene implants. Despite the fact that classic metal-on-conventional uncross-linked polyethene implants have been utilised for many years, this is a new development. In the near term, research suggests that utilising metal on tightly cross-linked polyethene couples may improve lifespan [4]. Hard-on-hard bearing surface supporters maintain that these implants provide a better wear profile, minimise osteolysis, and increase implant life [5,6]. Despite significant advances in wear reduction using cross-linked polyethene, this remains the case. Furthermore, hard-on-hard bearing combinations allow for the implantation of bigger diameter femoral heads. This improves the head-to-neck ratio, raises the jump height, and potentially increases range of motion, all while lowering the chance of dislocation [7].

In the metal-on-metal prosthesis made by DePuy in Warsaw, Indiana, an ultralarge-diameter metal modular cobalt-chromium femoral head is combined with a monoblock cobalt-chromium acetabular shell. The DePuy Articular Surface Replacement (ASR^TM^) is the name given to this gadget. The acetabular component of the ASR prosthesis is a nonhemispheric cup with thin hydroxyapatite-coated sides. The device was approved by the United States Food and Drug Administration in 2003. The device's improved range of motion, decreased potential dislocation rate, and appealing wear profile were significant selling elements during its extended marketing effort in the United States. The implant was made accessible in the United States and promoted using statistics from European markets on early survival (Depuy Surgeon Information Product Manual). DePuy voluntarily halted sales of the device in the United States in March 2010 after receiving an increasing number of product complaints. In August of the same year, the business issued an official global product recall. Patients who got ASR implants had a greater revision rate than the general population, according to orthopaedic doctors [8]. It was discovered that negative responses to the major reason for early failures were adverse responses to metal debris (ARMD) [9,10]. Due to these issues, people are increasingly selecting alternative-bearing surfaces such as metal on ultra-high molecular weight polyethene and ceramic on ultra-high molecular weight polyethene implants over metal on metal-bearing surfaces [11]. Patients who get MoM hip replacements are encouraged by regulatory organisations across the globe, including the MHRA in the United Kingdom, to undertake monitoring and follow-up.

Because of worries about the early performance of this design, our organisation ceased its usage of it after less than two years. The authors describe a retrospective analysis of our early clinical experience with 24 consecutive total hip arthroplasty procedures utilising the DePuy ASR implant. All of these treatments were carried out in our facility. We assessed the efficacy of this implant design based on patient-reported outcomes and the proportion of patients who had clinical failure. Furthermore, we looked at the blood cobalt and chromium levels of all individuals who had these implants.

## Materials and methods

A two-year retrospective research was conducted on patients admitted to our clinic for elective THR surgeries starting in January 2007 and finishing in December 2008. To find these individuals, the hospital's system for identifying patients based on their diagnoses and surgeries was used. The research comprised patients who underwent a single unilateral THR with the DePuy ASR hip prosthesis for osteoarthritis or hip fracture. Patients who had bilateral hip arthroplasty or hip implants other than the DePuy ASR prosthesis throughout the follow-up period were excluded from the research. Institute for Clinical Research Singapore General Hospital issued approval 2018/2384.

A recall was carried out in the first quarter of 2013, during which patients were given an MRI scan of the hip and blood tests to detect their serum ion levels. Clinical information was gathered by reviewing the patient's follow-up clinic records. The patient-reported outcome measures (PROM) utilised in the evaluations were the Oxford Hip Score (OHS), the Western Ontario and McMaster Universities Arthritis Index (WOMAC), and the 36-Item Short Form Survey (SF-36), as well as a history and clinical examination. The demographics (age, gender) and clinical details of all patients were extracted and statistically analysed, including serum ion levels, femoral head and acetabular cup sizes, presence of complications (loosening, pseudotumor, infection) postoperatively, need for revision, WOMAC scores, SF-36, and the OHS.

## Results

**Figure 1:**
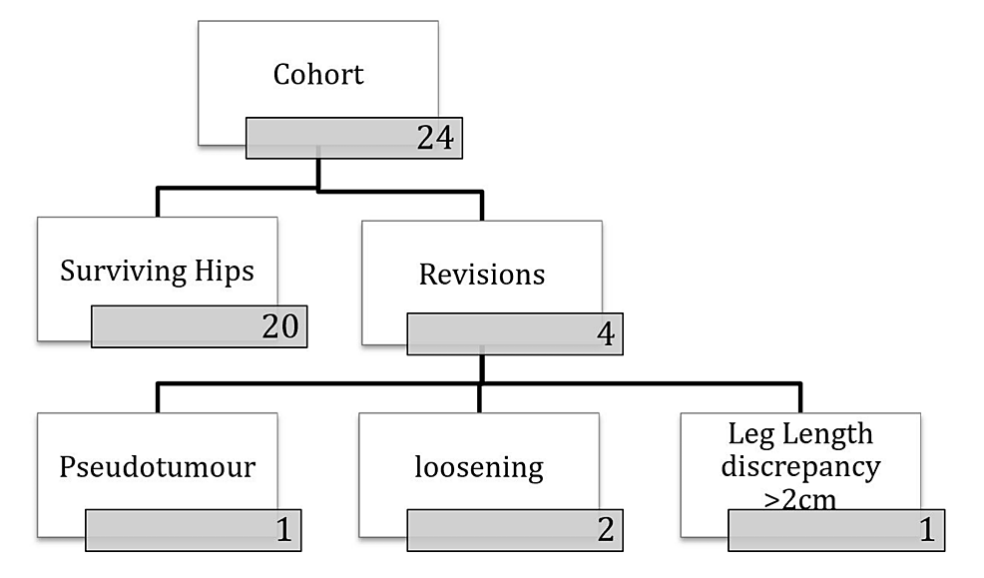
Our cohort patient distribution.

A total of 24 patients underwent elective unilateral hip surgery with the ASR hip system during the study period, giving a total of 24 ASR implant procedures for analysis (Figure 1).

Six resurfacing surgeries and 18 complete hip replacements made up the 24. Patients' mean age was 56.4 (SD 5.1). There were thirteen male patients (54.2%). Twelve (50%) of the main procedures were for osteoarthritis. Eight (66.7%) individuals with secondary osteoarthritis had hip avascular necrosis (AVN), one (8.3%) had slid capital femoral epiphysis, one (8.3%) had ankylosing spondylitis, and two (16.7%) had hip developmental dysplasia. The mean acetabular cup size was 53.0 mm, and the mean femoral head size was 47.1 mm.

WOMAC scores for the cohort showed statistically significant improvement five years post-op across all three domains of pain, disability and stiffness. SF-36 (Physical) and OHS also showed statistically significant improvement. There was no statistically significant improvement in SF-36 (Mental) score (Table 1).

**Table 1:** Clinical Outcome (Median (IQR)) WOMAC: Western Ontario and McMaster Universities Arthritis Index, SF36: 36-Item Short Form Survey, IQR: interquartile range

	Pre-op	5 years Post-op	p-Value
WOMAC (Pain)	58 (20)	100 (8)	p <0.05
WOMAC (Disability)	60 (45)	87.5 (30)	p = 0.033
WOMAC (Stiffness)	53.8 (27.7)	92.6 (18.8)	p < 0.05
SF36 (Physical)	37.5 (30)	80 (25)	p < 0.05
SF36 (Mental)	80 (20)	80 (24)	p = 0.673
Oxford	32.6 (10)	80 (24)	p < 0.05

Eight individuals had increased serum cobalt (5 g/L), averaging 22.5 g/L (range 0.5-257.7, SD 62.45). Eight individuals had high serum chromium (2 g/L) with a mean of 6.7 g/L (range 0.6-52.7, SD 13.08). Eight patients (33.3%) had MRI pseudotumors during follow-up.

Pain, leg length disparity, and implant loosening necessitated surgery in four (three females and one male) instances. Four intraoperative discoveries were unimportant; one was a pseudotumor. Our ASR cup revision rate is 16.7% because of two loosened acetabular cups and one leg length disparity (>2 cm).

## Discussion

The ASR implant recall served as a wake-up call to the orthopaedic and broader medical communities, particularly in terms of product approval for patient usage. Moving ahead, as the quality and design of MoM implants improve, we need to gather as much information as possible from our experiences with ASR implants. This increases doctors' awareness of perioperative issues. Recent research [12,13] has validated the higher failure rates linked with the DePuy metal-on-metal ASR system, particularly in large-diameter prosthesis resurfacing. These results stem from the Australian National Joint Replacement Registry's preliminary findings [14], which were published in 2005. This failure rate, followed by the subsequent recall of the implant, has piqued the curiosity of researchers [15]. One of the key concerns is the possible impact of Cr and Co debris accumulation, both locally and in the bloodstream. This prosthesis has been linked to three occurrences of cobalt poisoning [16,17]. One of the reasons this is one of the most troubling features is because of this.

After a five-year average follow-up period, our facility's revision rate was revealed to be 16.7%. At five years, the cumulative revision rate for ASR hip resurfacing system arthroplasties was 10.9% (95% CI, 8.7% to 13.6%), compared to 4% (95% CI, 3.7% to 4.5%) for all other resurfacing prostheses [14]. De Steiger et al. discovered this when reviewing the Australian National Registry. Despite the fact that this rate is high in and of itself, newer studies have shown findings that are similar to ours, with even higher rates at shorter follow-up periods. Bernthal et al., for example, found a three-year revision rate of 17.1% [13]. In their investigation, seven of the 12 implants that were effective during the first postoperative year had discomfort, three became loose, and one squeaked. All of these problems developed within three years, with two patients requiring a five-year follow-up. At five years, the cumulative revision rate for ASR hip resurfacing system arthroplasties was 10.9% (95% CI, 8.7% to 13.6%), compared to 4% (95% CI, 3.7% to 4.5%) for all other resurfacing prostheses [14]. Despite the lack of radiological evidence of loosening, the remaining patients reported of persistent pain. The radiographs of the patients in our series revealed no significant abnormalities, and the existence of chronic discomfort was the key criterion that necessitated revision. A variety of parameters, including component sizes, acetabular component anteversion and inclination, patient profile, and comorbidities, might explain the greater revision rate in our group. Our reduced femoral head size (average of 47 mm) contrasted with an average of 48-49 mm [18,19] in other Caucasian groups.

The size of the femoral head has a substantial impact on the wear rates of metal-on-metal implants. Greater components have been found in studies to form a thicker fluid layer between the articular surfaces [20] and to offer larger surface areas across which contact stresses are dispersed [9]. Langton et al. found considerably greater metal ion levels in individuals implanted with the smaller femoral component, indicating increased wear [9]. In accordance with these results, our cohort with a lower average femoral head size had greater revision rates than Caucasian cohorts. Although several other parameters, such as acetabular component orientation [9], contribute to implant wear, femoral head size should not be overlooked in the overall evaluation of implant wear analysis.

Although there is presently no agreement on whether quantities of metal ions in the circulation of patients implanted with orthopaedic implants are regarded acceptable or clearly hazardous, levels of cobalt below 2 g/L have been reported to be bearable [21]. In fact, regardless of the friction pair, these ions might be discharged from any implant. For example, in a study of 41 knee arthroplasties, chromium and cobalt levels in unilateral prosthesis were 0.92 and 3.28 g/L, respectively, whereas values in bilateral implants were 0.98 and 4.28, respectively [22]. There was also a case of cobalt poisoning in a patient who did not have a metal-on-metal implant at the time [23]. This patient was poisoned with cobalt.

Hip replacement surgery has continuously shown good results, and it was once dubbed the "procedure of the century" [24]. Many outcome scores, including the SF-36 and Oxford scores, have been developed to objectively analyse hip score results [25]. The Oxford, SF-36, and WOMAC ratings all revealed a considerable increase in how well the hip operated after five years.

## Conclusions

In conclusion, MoM hip arthroplasty had a greater revision incidence at five years in our group, presumably owing to the use of a smaller femoral head size. Nonetheless, MoM hip arthroplasty has the potential to be a long-lasting implant, and efforts should be made to enhance biomechanics and solve existing concerns, such as accelerated wear.
